# Metastable State of Water and Performance of Osmotically Driven Membrane Processes

**DOI:** 10.3390/membranes9030043

**Published:** 2019-03-22

**Authors:** Haifeng Zhang, Jie Wang, Ken Rainwater, Lianfa Song

**Affiliations:** 1 School of Chemical Engineering, Northeast Electric Power University, Jilin 132012, China; zhfeepu@163.com; 2 School of Environmental and Chemical Engineering, Tianjin Polytechnic University, Tianjin 300387, China; wangjie@tjpu.edu.cn; 3 Department of Civil, Environmental, and Construction Engineering, Texas Tech University, 911 Boston Ave, Lubbock, TX 79409-1023, USA; ken.rainwater@ttu.edu

**Keywords:** osmotically driven membrane processes, water flux, metastable state, cavitation, water continuity

## Abstract

Semipermeable membranes play critical roles in many natural and engineering systems. The osmotic pressure is found experimentally much less effective than the hydraulic pressure to drive water through the membrane, which is commonly attributed to the internal concentration polarization (ICP) in the porous layer of the membrane. In this study, it has been shown that a necessary condition for the osmotic pressure to be effective is water continuity across the entire membrane thickness under negative pressure, i.e., the water inside the membrane remains in a metastable state. However, the metastable state of water cannot be maintained indefinitely, and cavitation will undoubtedly occur in the osmotically driven processes. Collapse of the water metastable state was suggested for the first time to be a more important and fundamental reason for the low water fluxes in the osmotically driven membrane processes.

## 1. Introduction

Semipermeable membranes that allow water to pass but reject salts play critical roles in many natural and engineering systems [1,2]. Two major driving forces for water transport across the membrane are hydraulic pressure and osmotic pressure. Currently available membranes can produce sufficiently high water fluxes under reasonable hydraulic pressures so that potable water can be produced economically by desalination of seawater and brackish water. However, water fluxes through the same membranes under osmotic pressures of the same magnitude are only small fractions of these under hydraulic pressures [3,4]. Low water flux is a major obstacle for the practical applications of osmotically driven membrane processes, such as forward osmosis (FO) and pressure retarded osmosis (PRO) [5,6]. Understanding of the kinetic role of osmotic pressure on driving water across the membrane has been a long-lasting and serious challenge in this field [7].

Low water fluxes in the osmotically driven membrane processes are commonly attributed to the internal concentration polarization (ICP) developed in the porous support layer of the membranes [3,8,9]. It is postulated that the effective driving pressure on the active skin layer of a membrane can be significantly reduced by ICP in the porous support layer. A stronger ICP develops when the membrane porous support layer contacts with the solution than with water. Properties of the membrane porous support layer for ICP development can be characterized by the structural parameter S, which is a lumped parameter determined by the thickness, porosity, and tortuosity of the layer [5]. The stronger ICPs tend to develop in the support layers with the larger S values.

The semipermeable membranes originally developed for reverse osmosis (RO) are considered unsuitable for the osmotically driven processes because of the large S values. Therefore, one focus of research and development in this area was to make membranes with smaller structural parameters. Substantial progress has been made along this direction over the last four decades. Starting with the common RO membranes with S > 35,000 µm, new membranes with S < 500 µm have been specifically developed for the osmotically driven processes [10,11,12]. In spite of the much higher water fluxes obtained with the new generation of osmotic membranes in the laboratory settings, none of these membranes have been introduced into practical membrane modules for real-world applications.

Although the ICP-based theory may provide a seemingly reasonable explanation for low water fluxes in the osmotically driven membrane processes, it suffers seriously from the unreasonable values of the structure parameter. Different S values may be required for the same membranes to fit the observed experimental water fluxes under different osmotic pressures, which is against the original definition of the parameter that is an intrinsic property of a membrane. One more serious challenge or liability of the ICP-based theory is that S values used to fit the experimental data are neither measured directly nor determined by any other independent methods [13]. At this moment, the parameter S is practically used as no more than a fitting parameter to match the experimental water fluxes. Therefore, it is plausible that the overestimated ICP from an exaggerated S value masks or shadows other causes for the ineffectiveness of osmotic pressure on driving water across the membrane.

## 2. Negative Pressure and Water Metastability

**Figure 1:**
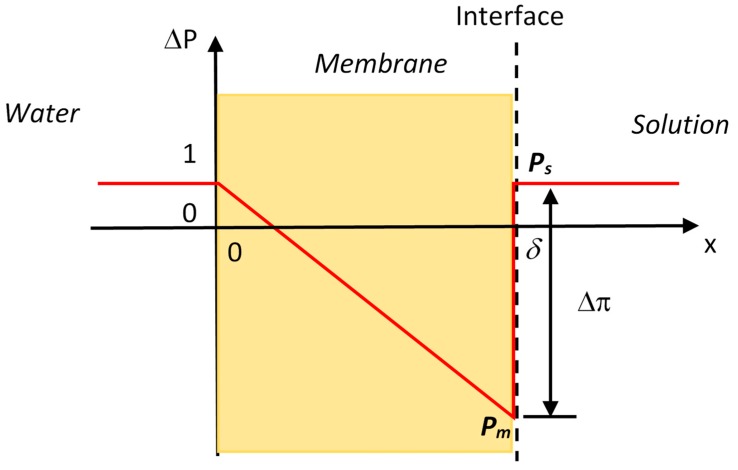
Hydraulic pressure profile along the membrane thickness induced by the osmotic pressure. Negative hydraulic pressure occurs inside of the membrane.

To understand water transport across the membrane under the osmotic pressure, the conditions for osmotic pressure to be effective need to be considered. The general driving force for water transport can always be presented as the difference in the chemical potential of water across the membrane. The isothermal chemical potential of water has two components that relate to the water activity and the hydraulic pressure, respectively:

(1) $$\mu =RT\ln a+P\bar{V}$$

where *μ* is the chemical potential of water, *R* is the gas constant, *T* is the absolute temperature, *a* is the water activity, *P* is the hydraulic pressure, and $\bar{V}$ is the molar volume of water. The chemical potentials of water in the membrane and in the solution are equal at the interface (Figure 1), i.e.,

(2) $$P_{m}\bar{V}=RT\ln a_{s}+P_{s}\bar{V}$$

where *P_m_* and *P_s_* are the hydraulic pressures in the membrane and in the solution at the interface, respectively, and *a*_s_ is the water activity in the solution. Because the solute cannot enter the semipermeable membrane, the activity of water is unity in the membrane and the activity term disappears on the left-hand side of Equation (2). The hydraulic pressure difference at the interface of membrane with the solution is then determined as

(3) $$\Delta P=\left(P_{m}-P_{s}\right)=\frac{RT}{\bar{V}}\ln a_{s}=-\Delta \pi$$

here the van’t Hoff equation is used to convert the water activity to the osmotic pressure in Equation (3). The hydraulic pressure on the solution side is usually small (1 atm when open to the atmosphere), the hydraulic pressure *P_m_* on the membrane surface usually falls into the negative range due to the relatively large osmotic pressures. Negative pressure means that water is stretched or under tension. More detailed discussions about the generation of negative hydraulic pressure at the interface of the membrane can be found in the literature [1,14,15]. The hydraulic pressure profile along the membrane thickness *δ* is schematically presented in Figure 1 when water and solution on both sides are open to the atmosphere. The hydraulic pressure is negative (i.e., water is under tension) for most of the membrane thickness and there is a jump in the hydraulic pressure at the interface of the membrane with the solution. Water actually moves as a bulk flow through the membrane driven by the hydraulic pressure gradient even the initial driving force is the osmotic pressure [16,17].

The osmotic pressure manifests itself as a negative hydraulic pressure at the interface of the membrane with the solution that pulls or draws water from the other side of the membrane. Therefore, water continuity across the entire membrane thickness is a necessary condition for the osmotic pressure to be effective for water transport across the membrane.

**Figure 2:**
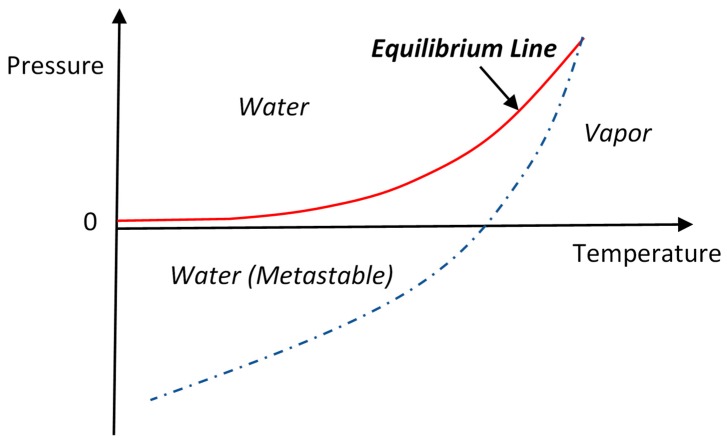
Schematic of phase diagram for water and the region of water in metastable state.

Can water continuity be maintained inside the membrane under a negative pressure? Yes, water can endure negative pressure without breakup of continuity under certain circumstances [18,19,20,21]. This feature is different than gases that have the smallest possible pressure of zero, i.e., the absolute vacuum. As shown in Figure 2, water and vapor can coexist at equilibrium only on a well-defined equilibrium line in the pressure-temperature plane [19]. Away from the equilibrium line, water is only stable above the line while vapor is only stable below. However, because of the liquid–vapor surface tension, if water is brought below the equilibrium line, it can maintain a liquid state for a finite time without vaporization. This special liquid state of water is called metastable state. The metastable region is schematically shown on Figure 2 between the solid equilibrium line and a broken line. The use of the broken line means that the lower boundary of the metastable region is not fixed but varies with experimental conditions.

However, the metastable state of water will eventually collapse due to bubble formation (cavitation) and return to the thermodynamically stable state of water and vapor co-existence, where the pressure is the vapor pressure at the given temperature along the equilibrium line. Water continuity breaks upon cavitation and, as a result, the osmotic pressure can no longer pull water across the membrane from the other side. Therefore, maintenance of the metastable state of water is essential for osmotic pressure to be an effective driving force for water transport across the membrane.

Water under negative pressure in the metastable state and cavitation inception in various types of waters have been extensively studied [19,20,21,22,23]. Except for specially treated ultrapure water of very small volume, waters can rarely retain a metastable state indefinitely because of the existence of cavitation nuclei in the forms of dissolved air, tiny air bubbles, and small solid particles. Cavitation often starts in such waters when pressure merely becomes negative [22,24,25,26,27]. Vapor bubbles form near propeller blades when the pressure just falls to about −1 atm [28]. In a well-controlled osmosis experiment, collapse of the metastable state of water was observed at a pressure of −2.4 atm even for an ultrapure water sample free of cavitation nuclei [15]. Therefore, cavitation is inevitable in the real-world membrane processes where the osmotic pressures are usually much higher than the threshold negative pressures for cavitation to occur in the processing water.

## 3. Model Development

**Figure 3:**
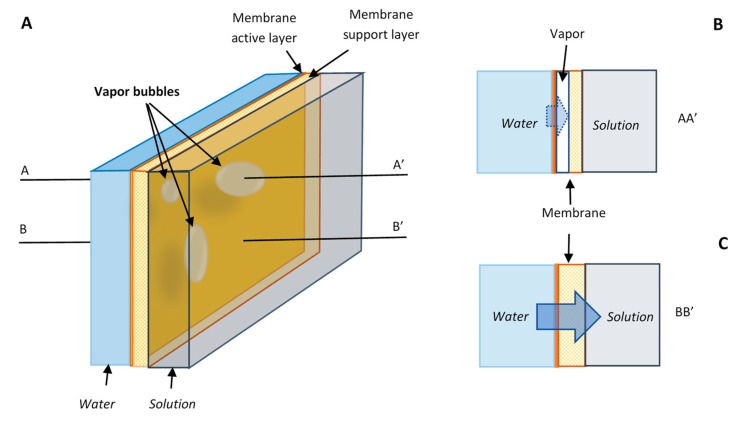
A schematic model for water transport under osmotic pressure. (**A**) Vapor bubbles can form and trapped in the porous support layer of a membrane. (**B**) The membrane area blocked by bubbles is unavailable or inefficient for water transport. (**C**) Water can only transport at full strength through the unblocked membrane surface.

A simple model is presented below to show the probable impact of cavitation on water flux in the osmotically driven membrane processes. Cavitation is most likely to occur inside the active membrane layer near the interface with the solution where the negative pressure maximizes. The porous support layer of membrane may help to trap and hold the vapor bubbles formed [29]. The vapor bubbles in the porous support layer of a membrane are schematically presented in Figure 3A. The membrane area that is blocked by the bubbles are no longer available for effective water transport under the osmotic pressure because water continuity is broken (Figure 3B). The osmotic pressure only works at full strength on the area free of vapor bubbles (Figure 3C). The sizes of the vapor bubbles in the figure are intentionally enlarged for illustration purpose. The bubbles or voids inside the membrane may not been obviously observed, and sometimes the membrane may be just simply dehydrated in some areas. The important point is that water continuity is broken somewhere inside of the membrane.

It is well established that the number and volume of vapor bubbles increase as pressure becomes more negative [22]. Therefore, the fraction of membrane area blocked by vapor bubbles increases with osmotic pressure. For illustration purpose, the fraction of membrane area blocked by vapor bubbles is expressed as

(4) $$\lambda =\lambda_{0}+\left(1-\lambda_{0}\right)\left(1-e^{-\alpha \Delta \pi }\right)$$

where λ is the fraction of membrane area blocked by vapor bubbles, $\lambda_{0}$ is the initial fraction of the blocked membrane area, α is a parameter to characterize the membrane process for cavitation, which can be a function of water quality, membrane material hydrophobicity, and other characteristics of the membrane process, and Δπ is the osmotic pressure of the solution. It is noted that λ = $\lambda_{0}$ when Δπ = 0 while λ → 1 as Δπ → ∞. Then the water flux on a partially blocked membrane can be calculated by

(5) $$J=\left(1-\lambda \right)J_{0}+\lambda J_{b}$$

where *J* is the overall water flux, $J_{0}$ is the water flux through the unblocked membrane area fraction, and $J_{b}$ is the water flux through the blocked membrane area fraction.

When both water and solution are open to the atmosphere, the net driving force in the bubble-blocked area is approximately 1 atm because vapor pressure is negligible at room temperature. Water flux in the bubble blocked area is usually negligible compared to the unblocked area where the net driving pressure is much greater than 1 atm. Therefore, for an initially completely wetted membrane ($\lambda_{0}$ = 0) and both water and solution open to atmosphere, Equations (4) and (5) can be combined to result in

(6) $$J=Ae^{-\alpha \Delta \pi }\Delta \pi$$

## 4. Discussion

**Figure 4:**
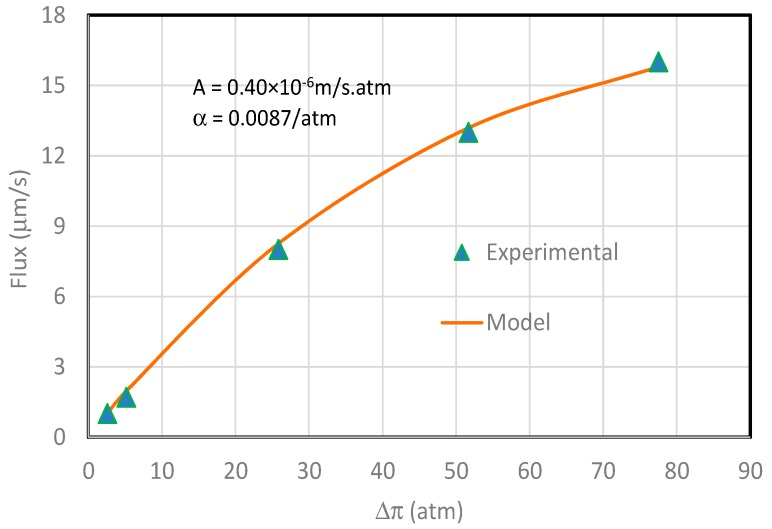
Modeled water flux (line) and experimental water flux (symbols) under osmotic pressure. The experimental data were taken from Figure 7 of Reference [30] and the parameters used in modeling are *A* = 0.40 μm/s⋅atm, and *α* = 0.0087/atm.

As a rapid communication, the usefulness of Equation (6) is briefly discussed by comparing model calculations with a group of the experimental water fluxes under osmotic pressure in the literature [30]. The experiment was conducted with a CTA membrane provided by Hydration Technologies, Inc. (Albany, OR, USA). Deionized water and sodium chloride solution were used in the FO experiment. The water fluxes were directly read from Figure 7 in the cited reference. The comparison is shown in Figure 4 with the symbols for the experimental water fluxes and the line for the model calculations. The figure shows that the model calculations fit experimental data extremely well when the parameters *A* = 0.40 × 10^−6^ µm/s⋅atm and *α* = 8.7 × 10^−3^/atm are used in the simulations.

This communication provides a completely new perspective on the fundamental reason for the ineffectiveness of the osmotic pressure as a driving force for water transport across the membrane. There are overwhelmingly abundant evidences that cavitation will readily occur in waters under the conditions like in the osmotically driven membrane processes. The metastable state of water cannot be maintained indefinitely in the processes to provide the needed water continuity for the osmotic pressure to pull or draw water effectively through the membrane. Therefore, a fundamental theory for water flux in the osmotically driven membrane processes cannot be complete without appropriately addressing the metastable state of water and its collapse in the processes.

Water metastability is not related to ICP in the membrane porous layer. Cavitation and ICP are two irrelevant phenomena in the osmotically driven membrane processes. The above claim on the importance of water metastability does not deny the possible impact of ICP on water flux in the osmotically driven membrane processes. However, the metastable state and water continuity are more essential and important to the performance of the osmotically driven membrane processes. Other factors, including ICP, may have to be reconsidered and realigned with an appropriate treatment of the metastable state of water for a better understanding of the kinetic role of the osmotic pressure on water transport across the membrane. By the way, because of the role the metastable state of water in the osmotically driven membrane processes, water flux in the processes is unlikely to be significantly increased by the modifications and improvements of membranes aimed to reduce ICP in the porous support layer of the membrane.

Unfortunately, this important mechanism controlling water flux in the osmotically driven membrane processes has never been considered before. Although this preliminary study strongly supports the role of water metastable state on the effectiveness of osmotic pressure as a driving force, more detailed and systematic investigations are needed to reconfirm the preliminary findings and to finally prove the hypotheses. Another interesting aspect to investigate is the factors that affect water metastability and cavitation in the processes, such as level of dissolved air in water and the hydraulic pressure applied on water side and/or solution side of the membrane. Systematic investigations are needed to study the osmotically driven membrane processes along this completely new and very promising direction.
